# Evolving Dynamics of Fermented Food Microbiota and the Gut Microenvironment: Strategic Pathways to Enhance Human Health

**DOI:** 10.3390/foods14132361

**Published:** 2025-07-03

**Authors:** Antonia Terpou, Divakar Dahiya, Poonam Singh Nigam

**Affiliations:** 1 Department of Agricultural Development, Agri-Food, and Natural Resources Management, School of Agricultural Development, Nutrition & Sustainability, National and Kapodistrian University of Athens, Evripos Campus, 34400 Evia, Greece; 2 Basingstoke and North Hampshire Hospital, Basingstoke RG24 9NA, UK; ddahiya@hotmail.co.uk; 3 Biomedical Sciences Research Institute, Ulster University, Coleraine BT52 1SA, UK; p.singh@ulster.ac.uk

**Keywords:** food–gut axis, health-promoting microbes, microbiome modulation, microbial metabolites, intestinal balance, disease modulation, nutritional health, immune system support

## Abstract

The growing interest in health-promoting diets has brought fermented foods into the spotlight due to their unique microbial compositions and bioactive metabolites. Fermented foods and their beneficial microbiota are expected to stimulate the overall industry’s expansion over the next few years as their beneficial health effects become established. This narrative review explores the evolving dynamics of fermented food microbiota and their interactions with the gut microenvironment, emphasizing strategic pathways to enhance human health. Fermented foods, both industrially produced and traditionally prepared, serve as carriers of beneficial microorganisms such as lactic acid bacteria, yeasts, and certain fungi that transform food substrates into bioactive compounds including short-chain fatty acids (SCFAs), exopolysaccharides, and bioactive peptides. Simultaneously, their bioactive metabolites are the subject of passionate investigation by the scientific community, uncovering novel beneficial aspects that have not been elucidated until now. These metabolites contribute to improved gut barrier function, modulation of immune responses, and overall metabolic health. Notably, microbial fermentation can reshape the intrinsic properties of food, offering therapeutic potential beyond basic nutrition. The interactions between food-derived microbes and the host gut microbiota suggest a synergistic mechanism influencing gastrointestinal and systemic health outcomes. Nevertheless, there remains a significant gap in the comprehensive evaluation of the existing literature in this specific research area. Further research is needed to standardize fermented food formulations, validate the effects of individual microbial strains, and optimize their application in personalized nutrition and functional food development. Accordingly, this review highlights the association between the microbiota of fermented foods and their metabolites with the gut microenvironment, emphasizing their potential health-promoting properties.

## 1. The Dynamic Interaction Between Consumption of Fermented Foods and Gut Microbiota

Growing interest in health-promoting diets has brought naturally derived, minimally processed foods to the forefront, with fermented products attracting particular attention for their potential functional benefits, including improved gut health, enhanced nutrient bioavailability, and modulation of the immune system [1,2]. Notably, fermented foods and their beneficial microflora have captured the attention of consumers, scientists, and the global industry alike, thanks to their microbially transformed metabolites which can exhibit therapeutic properties. Currently, fermented foods and beverages account for roughly one-third of the human diet [3]. Food fermentation is actually a biological method of food preservation, and it can be accomplished by introducing specific microbes under controlled conditions, which enables the bioconversion of food substrates through their growth and metabolic activities. Currently, industrial food fermentation is accomplished through the use of selected starter cultures, which are employed in large-scale production. As modern microbiology has advanced, specific starter cultures have been identified and selected to be applied for food fermentation, targeting desirable characteristics of food products. The microorganisms typically involved in food fermentation primarily include lactic acid bacteria, yeasts, bacilli, and filamentous fungi [2,3,4]. In local areas, there are still producers engaged in small-scale production who have preserved their “inherited starters” using traditional methods that have been passed down from one generation to the next. Traditional fermented food can also be crafted through spontaneous or back-slopped fermentation methods [1,4]. These practices serve as a valuable source of indigenous microorganisms with potential health benefits [4].

**Figure 1:**
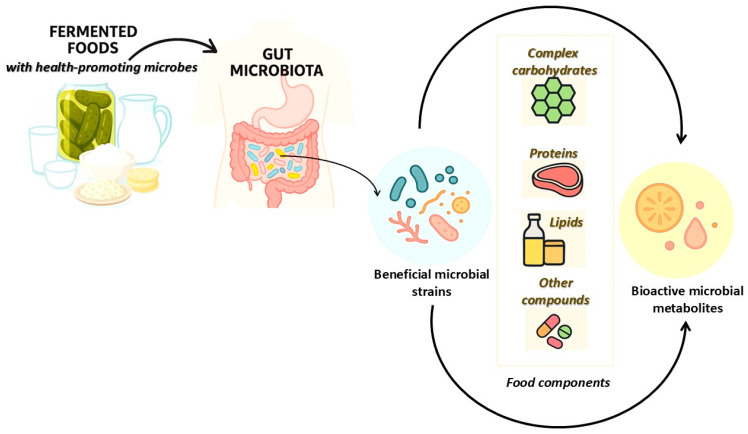
Simplified illustration of food compound transformation into bioactive metabolites after consumption of fermented foods with beneficial microbes.

Microbial fermentation can alter the intrinsic bioactive properties of food (Figure 1). Fermented foods, enriched with health-promoting microbes, serve as dietary sources of beneficial microbial strains, including lactic acid bacteria, yeasts, and other probiotics [3,5]. Upon ingestion, these microorganisms interact with the host gut microbiota, influencing microbial composition and metabolic activity [3]. Central to this interaction is the bioconversion of key food components such as complex carbohydrates, proteins, lipids, and other dietary compounds by both resident and introduced microbial strains [3,6,7,8]. These substrates are metabolized into a wide array of bioactive microbial metabolites, including short-chain fatty acids (SCFAs), bioactive peptides, exopolysaccharides, and vitamins [3]. This pathway highlights the therapeutic potential of fermented foods in modulating the gut microenvironment, enhancing nutrient bioavailability, and promoting systemic health benefits (Figure 1). For instance, it was reported recently that SCFAs produced as microbial metabolites have the capacity to modulate gut immune function by interacting with immune cells such as dendritic cells and T cells and by promoting the production of anti-inflammatory cytokines [9]. Furthermore, SCFAs such as butyrate may serve as a primary energy source for colonic epithelial cells, promoting barrier integrity and reducing gut permeability [10]. Beyond gut immunity, SCFAs and other microbial by-products have been implicated in regulating metabolic pathways, reducing oxidative stress, and influencing gut–brain axis signaling. These effects support emerging evidence linking fermented food consumption with improved cognitive function, stress resilience, and mental well-being [11,12,13]. Consequently, fermented foods not only deliver live beneficial microbes but also supply a reservoir of bioactive metabolites that exert local and systemic health effects [3]. Thus, fermented foods are highlighted not only as nutritional sources but also as functional vehicles for microbiota-driven bioactivity, underscoring the need for integrated research in diet and microbiome science to support functional food development and personalized nutrition.

In conclusion, the interplay between fermented food consumption and gut microbiota represents a promising avenue for enhancing human health through dietary modulation. Thus, this review focuses on the intricate relationship between fermented food consumption and the gut microenvironment, highlighting the health-promoting potential of both the microbial communities present in these foods and their bioactive metabolic by-products. Through microbial fermentation, food substrates are transformed into bioactive compounds, which have been shown to modulate immune responses, support gut barrier function, and influence systemic metabolic pathways. These mechanisms suggest a clear link between fermented foods and improved health outcomes, including gastrointestinal and immune health as well as mental well-being. To explore these evolving dynamics, a narrative literature search was conducted, focusing on the interactions between fermented food microbiota and the gut microenvironment, with an emphasis on strategic pathways to enhance human health. Relevant peer-reviewed articles, primarily from the last decade, were identified through structured searches in PubMed, Scopus, ScienceDirect, and Google Scholar using combinations of keywords such as “fermented foods”, “food microbiota”, “gut microbiota”, “gut microenvironment”, “probiotics”, “postbiotics”, and “human health”. Studies included in this review addressed the microbial composition of fermented foods, their influence on gut ecology, and associated health outcomes, while non-peer-reviewed sources were excluded. The synthesis of this literature supports the conclusion that fermented foods represent a promising, evidence-based approach to functional nutrition and health promotion, warranting further interdisciplinary research and innovation.

## 2. Fermentative Bioprocesses and Microbial Transformation of Food Substrates

Fermentative bioprocesses play a crucial role in food production, as microbial activity significantly affects both the composition and nutritional value of foods [3]. A wide variety of substrates derived from dairy and plant sources can be used for fermentation, including dairy milk, meat, fruits and vegetables, seafood, cereals, grains, root crops, and various other region-specific food products. For each type of food substrate, a specific group of microorganisms can be selected based on the sugar content of the substrate and the fermentation abilities of the microbial strains involved [1,14,15]. These microbial transformations begin during fermentation and often continue within the gastrointestinal tract after consumption, contributing to overall health benefits [3].

Food substrates rich in monosaccharides and disaccharides are usually subject to fermentation by yeasts or lactic acid bacteria. Conversely, food substrates abundant in polysaccharides are best fermented by diverse microbial populations, encompassing molds, yeasts, or bacilli. Specifically, hydrocarbons are valorized during microbial fermentation in cereal- and grain-based foods. Products such as sourdough and fermented millet depend on the activity of lactic acid bacteria and yeasts (e.g., *Saccharomyces cerevisiae*) to break down complex starches, releasing organic acids and carbon dioxide (CO_2_). These microbial transformations not only lower the glycemic index of the foods but also produce prebiotic compounds that promote the growth of beneficial gut bacteria [16,17,18]. Similarly, in dairy-based fermented foods (e.g., yogurt, kefir, and sour milk), lactose serves as the primary substrate. It is metabolized by *Lactobacillus* spp. and *Streptococcus thermophilus* into lactic acid, leading to acidification and enhanced digestibility [1,2]. Additionally, these microbes can produce bioactive peptides with antihypertensive or immunomodulatory effects, which may persist and be further metabolized by the host gut microbiota [19,20,21]. In vegetable-based fermentations such as kimchi and sauerkraut, fiber and plant-derived oligosaccharides are fermented by *Leuconostoc* spp., *Weissella* spp., and *Lactobacillus* spp., producing short-chain fatty acids (SCFAs), antimicrobial compounds, and vitamins such as folate and vitamin K2 [22,23]. These compounds are associated with improved gut barrier integrity and anti-inflammatory activity once absorbed in the colon [24]. Legume-based fermentations, like miso and natto, involve proteolytic microbes such as *Bacillus subtilis* and *Aspergillus oryzae*, which hydrolyze soybean proteins into bioactive peptides with antioxidant and lipid-lowering effects [25,26,27]. In meat and fish fermentations, protein-rich substrates are transformed by lactic acid bacteria, resulting in flavor-enhancing amino acids and antimicrobial peptides [28]. Some of these compounds, such as γ-aminobutyric acid (GABA), have been associated with blood pressure regulation and neuroprotective functions after gut absorption [29].

Furthermore, fermented foods are renowned for their abundance of antimicrobial substances, such as organic acids, ethanol, and bacteriocins, all of which are produced through microbial transformation either during food fermentation or within the gut microenvironment [3,5]. Another significant aspect of fermented foods is that their components can enter the human body either directly through interaction with beneficial live microorganisms or indirectly through the consumption of microbial metabolites produced during food fermentation [30]. In both cases, microbial activity interacting with food components can enhance the concentration of bioactive compounds. The fermentation of food raw materials can also take place in the gut, a bioprocess known as colonic fermentation. A key outcome of colonic fermentation is the increased bioavailability of various bioactive compounds, whose effectiveness often depends on the nature of the original food substrate. These microbially transformed metabolites are generally more potent in delivering therapeutic effects than their parent compounds [3,13,31].

Among health-promoting fermented foods, probiotic formulations are particularly notable, as they contain viable probiotic microorganisms or live biotherapeutic agents. Their regular consumption has been linked to a broad spectrum of clinically relevant health benefits [5,32]. These include prevention of necrotizing enterocolitis in infants, symptom relief for individuals with functional bowel disorders, reduced risk of antibiotic-associated diarrhea, improved management of ulcerative colitis, and decreased incidence and duration of common upper respiratory and gastrointestinal infections [5,31].

These foods offer enhanced benefits to consumers beyond those provided by essential nutritional components, including anticarcinogenic properties, and supplementation of vitamins and bioactive compounds [5,32]. Research indicates a correlation between reduced consumption of live microbes and the rising prevalence of modern autoimmune diseases [31]. This integrated perspective highlights that microbial fermentation is not confined to food processing alone; it continues in the gastrointestinal tract, where the microbial transformation of residual substrates contributes to a range of physiological effects. Consequently, substrate-specific fermentation processes and their microbial by-products should be considered not only for food safety and preservation, but also for their functional roles and health-promoting potential throughout the human digestive system [3,4,33].

## 3. Beneficial Microbes for Fermented Foods and Their Bioactive Metabolites

Archaeological evidence suggests that the process of food fermentation was discovered inadvertently thousands of years ago. Since the early days of civilization, fermented foods have been crafted through spontaneous fermentations, primarily aiming to preserve raw materials [1]. Fermented foods continue to play a significant role in human nutrition, garnering increasing attention. This is reinforced by evidence-based research indicating that live microbial cultures can provide health benefits to consumers [5].

Fermented foods are established as nutritionally and functionally rich foods produced by microbial action associated with bacteria, yeast, mycelial fungi, and their enzymes. Fermented foods can provide a diverse array of microorganisms, and their involvement in medicinal values can lead to a broad spectrum of health-promoting effects. The primary starter cultures used in the production of fermented foods include lactic acid bacteria (LAB) (e.g., *Lactobacillus* spp., *Streptococcus thermophilus*, *Bifidobacterium* spp., and several other probiotic bacterial species) and *Saccharomyces* in the cases of wine, beer, and bacteria plus yeast in sourdough bread production. According to numerous recent studies, the consumption of fermented foods containing live microbial cultures can yield a multitude of health advantages [5,12,14,31,34,35,36,37,38,39]. These may include the production of antimicrobial compounds targeting potential pathogenic microorganisms, as well as antimutagenic, anticarcinogenic, and antitumor effects [3,5,40,41,42,43,44].

The presence of specific lactic acid bacteria (LAB) and Bifidobacteria in both humans and animal models suggests that these microbial groups may have the potential to modulate host metabolism and immune function [3,21,45,46]. Similarly, there is documented evidence that *Saccharomyces* yeasts exhibit significant anti-inflammatory properties [47]. This aligns with observations that moderate alcohol consumption, often involving yeast-fermented beverages such as beer and wine, may be associated with certain immune-supporting effects. However, it is important to note that the relationship is complex, and excessive alcohol consumption has well-documented detrimental effects on the immune system. In addition, fermented foods may help improve metabolic and physiological disorders, lower cholesterol levels, enhance probiotic activity, and promote the production of exopolysaccharides (EPSs) that can serve as prebiotic ingredients [31]. They also contribute to the generation of a variety of other bioactive compounds with the potential to benefit overall consumer health [3].

In recent decades, the term “*Bioactive Food Component*” has emerged as a significant concept in scientific literature, aiming to describe food compounds capable of influencing biological processes or substrates, thereby affecting body functions or conditions, and ultimately, health [48]. These components have recently garnered extensive attention in research due to their perceived potential in promoting health and preventing diseases [33]. Similarly, intensive research is being conducted on the metabolic compounds produced by fermented food starter cultures to understand their functional properties [3,20,49].

Microbial metabolites can enhance fermented foods by introducing a multitude of bioactive components, which are well-documented in the recent literature for their potential to offer various health benefits to consumers [3,5,8]. Notably, today’s accurate analytical and biochemical research tools demonstrate the presence of various compounds providing biological activity in fermented food products [30]. Several recent studies and some placebo-controlled studies have provided evidence of the health-promoting effects of fermented foods (Table 1).

According to statistical data, global cheese consumption reached approximately 22 million tons in 2022. Consequently, cheese—particularly fermented cheese products that undergo extensive microbial aging or ripening—can contribute significantly to human health by offering a range of potential health benefits (Table 1). Worldwide, there are approximately 1500 different types of fermented cheese products produced annually, including gouda, feta, mozzarella, cheddar, roquefort, stilton, and gorgonzola, among others [28,29,30,31,32,33,34].

A noteworthy discovery highlighting the importance of incorporating fermented cheese products into one’s diet has emerged from a recent randomized controlled trial [70]. In this trial, the influence of individual components of cheese was evaluated when consumed either entirely or partially within a fermented cheese matrix during a real-life intervention. For this investigation, Irish cheddar cheese, a high-fat fermented dairy product, was chosen by the researchers to examine the impact of high-fat consumption on blood lipid levels. The findings appear to validate that, even when consumed in substantial quantities, dairy fat within the cheese matrix does not negatively affect the blood lipid profiles of individuals at risk of metabolic diseases [70]

Regular consumption of sour milk has been demonstrated to have a notably significant effect on reducing blood pressure in various studies [56,57]. In particular, a recent study demonstrated the antihypertensive effects of sour milk among hypertensive participants who were not taking antihypertensive medications. Significantly, the systolic blood pressure of these participants exhibited a notable reduction after 2 and 4 weeks of sour milk consumption when compared to the placebo group that consumed unfermented acidified milk [57].

Another type of fermented food with great popularity in Asia involves soybean fermentation. Research has indicated that soybean extracts possess anticancer properties, which can lead to lower cholesterol levels and have antihypertensive effects in both spontaneously hypertensive rats and humans [37,38,39,40]. Recent studies have shown that long-term consumption of miso soup can help mitigate salt-induced hypertension. Miso soup is a classic Japanese dish that starts with a dashi stock and is seasoned with miso paste. The production of miso involves a two-stage fermentation process. In the first stage, a mold such as *Aspergillus oryzae* is introduced to a substrate to create koji. In the second stage, bacteria and yeast are added as the koji is mixed with a salt and soybean mash. The miso is then allowed to ferment for a period of up to 2 years. [85].

The production of beer and wine played a significant, and even crucial, role in the advancement of agriculture and the shift of human societies from nomadic hunter-gatherer communities to settled farming civilizations. Palm wine presents a range of health advantages, making it a compelling and noteworthy beverage deserving of microbiota analysis. The presence of acetic acid bacteria (AAB), yeasts, and LAB, contributes to lactic, alcoholic, and acetic acid fermentation in palm sap. Prior research has employed microbiological methods to explore the microorganisms within palm wine and has identified antibacterial properties effective against certain infections [83]. The ecological factors of the microbial community affect its metabolic activities and, consequently, the composition of the fermented product.

## 4. Fermentation-Driven Bioactive Compounds for Functional Food Applications

Microorganisms have been integral to food production since the earliest stages of human civilization, offering one of the most cost-effective and sustainable methods for food processing and preservation. Traditionally, fermentation processes were driven by spontaneous microbial activity originating from raw materials, which harbored complex, naturally occurring, and often taxonomically undefined microbial consortia [1,4,86]. Over time, the use of selected starter cultures has allowed for greater control, consistency, and predictability in fermentation outcomes. However, many traditional fermented foods are still produced through spontaneous fermentation, without deliberate microbial inoculation [2,23]. It is important to note that while some modern fermented products are processed in a way that eliminates viable microorganisms from the final product, the majority of widely consumed fermented foods such as yogurt, sour milk, kefir, cheese, kimchi, kombucha, dry fermented sausages, and miso retain substantial populations of live microbes. These often range from 10^6^ to 10^9^ colony-forming units per gram (CFU/g) [2,5,26,87,88,89,90]. Traditional fermentation practices include both spontaneous and back-slopped fermentation. In back-slopping, a portion of a previous successful fermentation (e.g., yogurt, whey, or sourdough) is used to initiate the next batch, allowing for the enrichment of a resilient and functionally adapted microbial community.

In industrial fermentation, the use of standardized starter cultures is essential to ensure the quality, safety, and consistency of the final product. These microbial cultures, primarily composed of lactic acid bacteria (LAB) and yeasts, drive the fermentation process by metabolizing sugars into lactic acid or ethanol [91,92,93]. This metabolic activity initiates a series of biochemical transformations that not only influence the flavor, texture, and preservation of fermented foods but also lead to the generation of bioactive components with significant functional and health-promoting effects.

**Figure 2:**
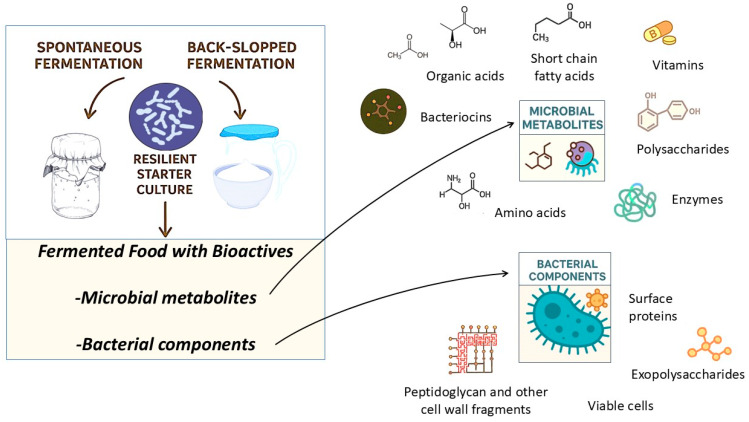
Bioactive compounds present in fermented foods as a result of beneficial microbial activity during controlled or spontaneous fermentation.

As illustrated in Figure 2, the bioactive components of fermented foods can be broadly categorized into two main groups: (1) bacterial components and (2) microbial metabolites [94]. Bacterial components refer to structural and functional elements of the microorganisms themselves, such as cell wall fragments, surface proteins, exopolysaccharides, and viable probiotic cells. These elements can directly interact with the host’s immune system and gastrointestinal environment, influencing various physiological responses. In contrast, microbial metabolites are low molecular weight compounds synthesized either during food fermentation processes or as a result of microbial activity within the gut [5,94]. These bioactive components in fermented food may include short-chain fatty acids, organic acids, bioactive peptides, bacteriocins, amino acids, vitamins, and various other functional molecules. Collectively, these compounds, arising from both microbial cell structures and metabolic by-products, form the basis of the health-promoting effects associated with fermented foods. Their presence underscores the pivotal role of microbial fermentation not only in enhancing food safety and preservation but also in driving the development of functional food products with targeted physiological benefits [3,95,96,97].

### 4.1. Bacteria and Yeasts for Fermentation

Widely recognized as a model eukaryotic microorganism, *S. cerevisiae* plays a crucial role in the sugar and alcohol production, brewing, and baking industries due to its significant economic value. This commonly applied yeast strain serves as a valuable source of proteins, B vitamins, nucleic acids, and minerals, including a biologically active form of chromium referred to as glucose tolerance factor [90]. 

Literature reports verify the capacity of yeasts to generate extracellular peptides that exhibit inhibitory effects on both Gram-positive and Gram-negative bacteria as well as some virus species [40,98]. These peptides serve as antimicrobial components and are oligopeptides with varying sequence lengths, typically ranging from 10 to 100 amino acids [99]. They are released by yeast cells as they proliferate during the fermentation process. Antimicrobial peptides can be found in various living kingdoms, including bacteria, yeasts, fungi, and plants. They are present in many different fermented food products and act against various pathogenic or spoilage microorganisms, providing an immunomodulation response to the consumer [100,101].

Yeasts, being capable of releasing antimicrobial peptides, can effectively manage the proliferation of spoilage microorganisms throughout the fermentation process, thus diminishing the necessity for chemical antiseptics [98]. Nevertheless, these yeasts may also exert their inhibitory effects on beneficial microorganisms. Therefore, when employing yeasts or yeast-derived peptides as bio-preservatives, it is crucial to exercise selectivity to prevent any interference with the fermentation process caused by the inhibition of beneficial microorganisms [99].

Currently, the primary applications of *S. cerevisiae* biomass include its use in human and animal nutrition, the production of flavoring agents, by-product valorization, and as a filtering element for the clarification of beverages [102]. As *Saccharomyces* biomass is the second most significant by-product of the brewing industry, scientific studies have investigated the potential use of *S. cerevisiae* biomass in various biotechnological applications, including the extraction of valuable compounds and the biosorption of bioactive substances [103].

### 4.2. Microbially Derived Health-Promoting Metabolites in Fermented Foods

Microorganisms in fermented foods play a vital role in producing a wide array of health-promoting metabolites with significant functional potential. The nutritive compounds of a food matrix may alter via microbial transformation during food fermentation, providing novel bioactive compounds in the final food product [3]. As summarized in Table 2, the bioactive components found in fermented foods can be broadly classified into two categories: bacterial components, such as cell wall structures, surface proteins, and viable probiotic cells, and microbial metabolites, including short-chain fatty acids, organic acids, amino acids, vitamins, and other small molecules. Together, these compounds exert diverse physiological effects, ranging from immune modulation and gut microbiota balance to metabolic regulation and neuromodulation [3,5,58,104,105].

Among microbial metabolites, organic acids such as lactic acid and acetic acid play a central role in both acidifying and preserving fermented products. Beyond extending shelf life, they exert antimicrobial effects by inhibiting the growth of pathogenic and spoilage microorganisms, thereby enhancing food safety [5,96]. Alongside organic acids, bacteriocins, which are antimicrobial peptides synthesized by certain lactic acid bacteria, also contribute to the inhibition of pathogenic and spoilage organisms, thus supporting the stability of the gut microbiota and improving the safety of fermented food products. Microorganisms like *Lactococcus lactis* and *Lactobacillus* spp. produce bacteriocins, natural antimicrobial compounds that inhibit pathogenic bacteria and contribute to the safety of fermented products [115].

Fermented foods are also valuable sources of essential micronutrients. The fermentation process enhances the biosynthesis and bioavailability of B-complex vitamins, including B2 (riboflavin), B9 (folate), and B12 (cobalamin), which are critical for energy metabolism, red blood cell formation, and neurological function. These vitamins are often deficient in plant-based diets, making fermented foods an important dietary source [4,20,116].

Exopolysaccharides (EPSs) synthesized by strains such as *Lactiplantibacillus plantarum* (formerly *Lactobacillus plantarum*) and *Streptococcus thermophilus* also play multifunctional roles [21]. EPSs act as prebiotics, support immune modulation, and may help lower serum cholesterol levels. In addition, they contribute to the sensory qualities of fermented products, particularly texture and viscosity, enhancing both product quality and consumer acceptance [21,117].

Postbiotics represent another category of interest. These are non-viable microbial products or metabolic by-products released during fermentation or following the lysis of probiotic bacteria. While they do not contain live microorganisms, postbiotics retain significant bioactivity, including anti-inflammatory, antioxidant, and immune-regulating effects, and offer advantages in stability and safety compared to live cultures [20,118].

Bioactive peptides released during microbial proteolysis of dietary proteins demonstrate a wide range of physiological functions, including antihypertensive, antioxidant, antimicrobial, and immunomodulatory effects. These peptides are increasingly recognized as important contributors to the health-promoting properties of fermented foods [100]. Other essential metabolites include amino acids, which are either released or synthesized during fermentation. These compounds serve not only as building blocks for proteins but also as precursors for neurotransmitters and other bioactive molecules, thereby participating in important metabolic and neuromodulatory pathways.

Among microbial metabolites, short-chain fatty acids (SCFAs) such as acetate, propionate, and butyrate are among the most extensively studied microbial metabolites. Primarily produced by LAB and *Bifidobacterium* spp. through carbohydrate fermentation, SCFAs provide energy to colonocytes, reinforce gut barrier integrity, modulate immune responses, and are associated with reduced risk of colorectal cancer and metabolic disorders [44,119].

In addition, microbial fermentation can lead to the production of neuroactive compounds such as γ-aminobutyric acid (GABA), synthesized by LAB and lactococci. GABA is known for its neuroprotective, antihypertensive, and anxiolytic properties, and may play a role in gut–brain axis regulation [114]. Similarly, certain strains such as *Lactobacillus acidophilus* and *Bifidobacterium* spp. contribute to the production of conjugated linoleic acid (CLA), a bioactive lipid with demonstrated anticancer, anti-atherosclerotic, and immunomodulatory effects [43].

Collectively, these bioactive metabolites exemplify the biochemical complexity and functional diversity of microbial fermentation. Their occurrence in fermented foods highlights the capacity of fermentation processes to not only improve preservation and sensory attributes but also to modulate host physiology and contribute to human health through multiple well-defined biological pathways.

## 5. Impact of Diet on Sustaining Diversity in Gut Microbiota

The gastrointestinal tract houses an intricate ecosystem of microbiota. Within this ecosystem, the gut microbiota engages in intricate molecular interactions with the host, exerting influences on nutrition, immunity, and metabolism across an individual’s lifetime. Approximately 1.5 kg of bacteria inhabit the colon, with a density of 10^12^ cells per gram of intestinal content, establishing the gut microbiota’s recognition as a vital metabolic organ [14]. Trillions of microorganisms coexist in the adult intestine, capable of autonomously maintaining their own balance.

Over the course of long-term evolution, the gut microbiota has established a dynamic equilibrium with humans and plays a crucial role in preserving human physiological function and energy metabolism [10]. An increasing body of research has demonstrated that healthy microbial communities contribute to enhancing nutrient absorption, fortifying biological barriers, and regulating immune responses. However, when subjected to multifaceted internal or external environmental changes, the equilibrium of the normal gut microbiota can be disrupted, leading to physiological dysfunction and even a range of metabolic disorders [120].

Diet is recognized as one of the primary modulators of the composition and functional capacity of the human microbiome [121]. A strong body of evidence supports the relationship between long-term dietary patterns and gut microbial diversity, taxonomic structure, and gene functional profiles within the microbiome [122,123]. Observational studies have shown that fiber-rich, plant-based diets such as the Mediterranean diet are associated with greater microbial diversity and increased abundance of beneficial taxa, including short-chain fatty acid (SCFA)-producing bacteria. Moreover, controlled dietary intervention trials have demonstrated that even short-term dietary modifications can induce rapid and measurable shifts in the composition and activity of the gut microbiota, underscoring the dynamic and responsive nature of the microbial ecosystem to dietary inputs [6,108,123,124].

## 6. Contribution of Dietary Microbiota in Alleviating Diseases

A recent study proposed that a fermented food diet increases microbiome diversity and decreases inflammatory signals, whereas a high-fiber diet changes microbiome function and elicits personalized immune responses [125]. Certain fermented food products, such as kombucha, yogurt, kefir, and kimchi are widely consumed due to their reported associations with weight management and reduced risks of diabetes, cancer, and cardiovascular diseases [11,126,127].

The microbiota and fecal metabolome presented significant differences among fermented food consumers versus non-consumers in a longitudinal study [128]. It was observed that the metabolome of fermented food consumers was enriched with conjugated linoleic acid (CLA) [128]. CLA, an animal-source fatty acid, has been characterized as an anti-obesogenic, anticarcinogenic, and anti-atherosclerotic agent [129]. Fermented food consumption might serve as a valuable means to reestablish evolutionarily significant connections. Moreover, it could potentially reintroduce benign environmental and foodborne microbes that have been depleted due to the sterilization of the industrialized environment.

Consumption of dairy can be protective against type 2 diabetes (T2D) risk in in older adults at high cardiovascular risk [130,131,132]. In addition, consumption of fermented milk supplementation improves glucose metabolism and alleviates the effects of muscle soreness after high-intensity exercise [133]. Consumption of different fermented foods, such as fermented milk products with probiotics, is associated with alterations in brain intrinsic connectivity and has a protective effect against social anxiety symptoms and neuroticism [59,134].

By employing a combination of quantitative metagenomics, in silico genome reconstruction, and metabolic modeling, it was found that the consumption of a fermented milk product containing dairy starters and *Bifidobacterium animalis* enhances the production of colonic short-chain fatty acids [46]. Additionally, this dietary approach led to a reduction in the abundance of *Bilophila wadsworthia* compared to individuals with irritable bowel syndrome who consumed a regular milk product [46]. Consumption of probiotic fermented milk increased the abundance of *Bacteroidetes*, including members of the *Bacteroidaceae* or *Prevotellaceae* families, which are decreased during the non-ingestion period [134]. So, probiotic fermented milk has the potential to modify the microbial community structure in the gastrointestinal tract of adult humans while preserving the overall stability of the microbiota [134].

Kefir grains include microbial species *Lactobacillus brevis*, *L. paracasei*, *L. helveticus*, *L. kefiranofaciens*, *L. plantarum*, *L. kefiri*, *Lactococcus lactis*, *Streptococcus thermophilus*, *Acetobacter lovaniensis*, *Acetobacter orientalis*, *S. cerevisiae*, *S. unisporus*, *Candida kefyr*, *Kluyveromyces marxianus*, and *Leuconostoc mesenteroides* with antimicrobial activity against *Candida albicans*, *Salmonella typhi*, *Salmonella enterica*, *Shigella sonnei*, *Escherichia coli*, *Bacillus subtilis*, *Enterococcus faecalis,* and *Staphylococcus aureus* [42,60,135]. Strains isolated from kefir have been shown to colonize the human gut [39], leading to increases in the concentrations of *Lactobacillus* spp., *Lactococcus* spp., and *Bifidobacterium* spp. along with reductions in Proteobacteria and Enterobacteriaceae [35].

Recent studies have reported that components of kefir may modulate the immune system by suppressing viral infections, including those caused by the Zika virus, hepatitis C virus, influenza virus, and rotaviruses. In the context of COVID-19, kefir demonstrated antimicrobial and immunomodulatory activity by downregulating the expression of pro-inflammatory cytokines such as interleukin-6 (IL-6), interleukin-1 (IL-1), tumor necrosis factor-alpha (TNF-α), and interferon-gamma (IFN-γ) [61]. Furthermore, chronic administration of kefir in a rat model significantly reduced hypertension, along with marked decreases in tachycardia and left ventricular hypertrophy [136,137]. In similar experimental models, administration of probiotic strains such as *Lactobacillus fermentum* and *Lactobacillus coryniformis* in combination with *Lactobacillus gasseri*, *Lactobacillus helveticus*, *Lactobacillus paracasei*, and *Lactococcus lactis* has also demonstrated hypotensive effects, suggesting a potential role for these strains in cardiovascular regulation [52,138,139,140].

In the case of non-dairy fermented foods, a comparative analysis of three Kombucha consortia revealed that microorganisms such as *Komagataeibacter* spp., *Gluconacetobacter* spp., and *Gluconobacter* spp., along with yeasts like *Brettanomyces* spp. and *Schizosaccharomyces* spp., were among the most dominant. Furthermore, the metabolic profile was strongly correlated with the microbial composition [127]. Metabolites, such as caffeine, propanoic acid, and 2,3 butanediol differed greatly across the three kombuchas [127]. The consumption of kimchi demonstrated positive impacts on glucose metabolism-related factors and anthropometric measures in individuals with prediabetes. Fermented kimchi also exhibited additional benefits on blood pressure and insulin resistance/sensitivity as well as improvement in glucose tolerance [141]. In contrast to fresh kimchi, fermented kimchi directly modulates the gene expression of molecules involved in metabolic and immune pathways, or indirectly through alterations in gut microbial composition such as a decrease in *Blautia* and an increase in *Prevotella* and *Bacteroides* [142].

Inflammatory bowel disease (IBD) is associated with genetic, infectious, immunological, and environmental factors, including modification of the gut microbiota [7,143]. IBD, as typified by diseases such as ulcerative colitis (UC) and Crohn’s disease, affects the gastrointestinal tract without any satisfactory therapeutic approach. A diet with fermented foods has been suggested lately because of their anti-inflammatory properties for restoring the balance between helpful and harmful bacteria population in the gut [144]. This dysregulated microbiota composition is caused by modern environmental change, accompanied by lack of dietary fiber and prebiotics. Dysbiosis, the dysregulated microbiota composition, increases the inflammatory responses and increases the incidence of IBD [145,146].

Improvement of resolution in microbiome analysis revealed a reduction of the population of the bacterial phyla *Firmicutes* and *Bacteroidetes* but the upregulation of *Actinobacteria* and *Proteobacteria* during IBD [147]. In addition, fermented plant extract supplementation induces the anti-inflammatory *Firmicutes* phylum and *Clostridiales* order [148]. In Crohn’s disease patients, it has been observed that dietary yeasts cause a hyperinflammatory response from a subset of Th1 cells that are able to lyse the epithelium [149]. *Lactobacillus paracasei* TK1501-fermented soybeans alleviate dextran sulfate sodium (DSS)-induced colitis by the introduction of prebiotic metabolite lipoteichoic acid and peptidoglycan [49]. Kefir, a fermented milk product, has shown beneficial effects in a dextran sulfate sodium (DSS)-induced colitis model by reducing neutrophil infiltration and reticulum edema, as well as increasing the formation of autophagosomes [38]. Building on such findings, growing evidence supports the role of fermented diets in managing inflammatory bowel disease (IBD), prompting the launch of an ongoing clinical trial (NCT04401605). This trial investigates the impact of a diet enriched with fermented foods on inflammation and quality of life in patients with mild to moderate ulcerative colitis (UC). While early results are encouraging, indicating potential modulation of inflammatory cytokines and enhancement of gut microbiota diversity in healthy individuals [150], comprehensive clinical evidence remains limited. Notably, fermented plant extract (FPE) supplementation has been shown to increase the abundance of *Clostridiales*, a bacterial group associated with anti-inflammatory effects, and contribute to the suppression of intestinal inflammation [148]. Collectively, these findings underscore the promising therapeutic potential of fermented foods and their bioactive components in modulating gut health and managing chronic intestinal disorders.

## 7. Limitations and Controversies in the Fermented Food–Gut Axis

While fermented foods have gained widespread attention for their potential health benefits, several limitations and controversies within this field warrant critical consideration. One of the primary challenges lies in the variability of microbial content among fermented food products [2]. The microbial composition can vary significantly depending on factors such as the raw ingredients, fermentation conditions, starter cultures, and even geographic origin. This variability complicates efforts to standardize products and assess their reproducible health effects across populations [31,151]. For instance, traditional fermentation relies on spontaneous microbial communities, which are not always reproducible or clearly defined, raising concerns about batch-to-batch consistency and safety [13,93,152,153]. While these practices preserve cultural and microbial diversity, they may also carry risks related to inconsistent microbial viability, contamination, or the presence of opportunistic pathogens [4].

A significant limitation is faced regarding in vitro and animal studies which have demonstrated the highly promising effects of fermented foods such as immunomodulation, gut barrier protection, and metabolic regulation, while the translation to human clinical outcomes is still inconsistent. Many human trials suffer from small sample size, short duration, or lack of standardization in fermented product formulation, leading to mixed or inconclusive results. Systematic reviews often report limited evidence for cause–effect relationships due to confounding variables such as baseline diet, microbiota composition, and genetic factors [154,155].

Another area of controversy is the lack of consensus on viable microbial dose thresholds, probiotic strain specificity, and survivability of microbes through the gastrointestinal tract [5,151,156,157]. Even when viable microbes are present in fermented foods, not all strains colonize the gut or exert measurable effects, and their metabolic interactions with host cells remain only partially understood. The emerging concept of postbiotic non-viable microbial products or metabolic by-products adds complexity to how benefits are attributed, further blurring the lines between live microbial action and metabolite-mediated effects [5,10,118,154]. Additionally, there are safety considerations, particularly for immunocompromised individuals, the elderly, or those with gastrointestinal disorders [10,12,32,158,159]. While most fermented foods are considered safe, rare cases of contamination or adverse reactions due to uncontrolled fermentation or opportunistic microbes have been reported.

Regulatory inconsistencies across countries regarding labeling, health claims, and microbial content in fermented foods pose significant challenges for scientific validation and consumer trust [157]. The lack of global regulatory frameworks for defining probiotics and fermented products with functional claims limits the integration of these foods into evidence-based nutritional guidelines [159].

Overall, addressing these limitations will require interdisciplinary efforts, standardized research protocols, and high-quality clinical trials to clarify the functional mechanisms of fermented foods and reliably translate them into public health strategies.

## 8. Concluding Remarks and Future Strategies

The human gut microbiome is composed of trillions of microorganisms, and changes in the composition and activity of these resident microbes have been associated with a range of systemic diseases, particularly those involving the immune system [120]. Recent research highlights that the microbiota of individuals in industrialized societies differs markedly from that of our recent ancestors [160]. Rapid modernization, including medical interventions and dietary changes, has contributed to the progressive degradation of the microbiota, a process thought to be implicated in the rising incidence of various diseases common in industrialized populations. Meanwhile, accumulating evidence underscores a bidirectional relationship between fermented food products and the gut microbiota. The gut microbiota metabolizes natural compounds, leading to the production of metabolites that vary in bioactivity, biotransformation, and toxicity.

The growing interest in the human microbiome as a critical determinant of human health and behavior underscores the need to understand the functions of microorganisms and their metabolic by-products introduced into the gastrointestinal tract through dietary intake. Fermented foods are now recognized for their attributes that extend well-being beyond simple preservation and sensory attributes. With the development of microecology and medicine, gut microbiota has been reported to play an important role in the pharmacological effects of food products; besides, fermented foods have also been demonstrated to be effective for modulating the gut ecosystem and maintaining the balance. However, it is also important that the probiotic products, including fermented food and functional beverages, produced from perishable dairy milk or non-dairy sources, should be available to consumers in the functional state of cultures contained in the products. Therefore, the use of the right quality of packaging materials is very important to retain the viability and activity of probiotic cells during transportation of fermented products to the shops and also for stocking them with a reasonable shelf life [161].

With the rapid advancement of meta-omics technologies, the field is moving closer to implementing true systems biology approaches in nutrition and microbiome research. Future research should focus on leveraging these tools to analyze the co-variation between microbial community structures and their metabolic outputs under varying dietary conditions, thereby identifying specific microbial strains and metabolites that are directly linked to measurable health outcomes. A deeper understanding of the complex interactions among dietary components, food-associated microbes, the gastrointestinal microbiota, and host metabolism will enable the rational design of functional foods. In this context, fermented foods represent an “upstream” extension of the digestive process and provide a promising platform to deliver targeted health benefits through controlled microbial activity. Key future strategies include conducting strain-level analyses of fermentation-associated microbes to determine their functional traits, developing fermented food formulations enriched with well-characterized probiotics, and standardizing production and packaging methods to preserve microbial viability and activity. Furthermore, integrating microbiology, nutritional science, and systems biology within interdisciplinary research frameworks will be essential to support personalized dietary interventions based on individual microbiome profiles. Advancing such strain-specific and function-driven innovations in fermented foods will ultimately contribute to the development of next-generation nutritional solutions for health promotion and disease prevention.

## Figures and Tables

**Table 1:** Fermented foods and their starter cultures providing health-beneficial effects.

Fermented Food	Starter Cultures Causing Possible Bioactivity	Health-Beneficial Effects	Reference
Natural Yoghurt	*Lactobacillus delbrueckii* subsp. *bulgaricus* *Streptococcus thermophilus*	ACE-inhibitory	[50]
Yoghurt with probiotics	*Lactobacillus helveticus* *Lacticaseibacillus rhamnosus* *Lacticaseibacillus fermentum* *Bifidobacterium animalis* subsp. *lactis*	Antiproliferative, anticancer, immunomodulatory, antimicrobial, prevention of dysbiosis-associated weight loss, reduction of systemic inflammation, decreased prevalence of diabetic kidney disease, and promotion of oral health	[51,52,53,54,55]
Probiotic food beverages	*Lactobacillus delbrueckii* subsp. *bulgaricus* *Streptococcus thermophilus*, *Lactobacillus casei* *Bifidobacterium lactis* and many other species	For cognitive treatment via gut–brain signaling	[19]
Sour milk	*Lactobacillus helveticus* *Levilactobacillus brevis*	Antihypertensive effects, relieves anxiety, improves sleep quality	[56,57,58]
Fermented dairy and non-dairy products	*Bifidobacterium animalis* *Streptococcus thermophilus* *Lactobacillus delbrueckii* subsp. *bulgaricus*, *Lactococcus lactis*	Alleviating allergic reactions and symptoms	[59]
Milk-based or plant-sourced kefir	Probiotic LAB and *Bifidobacteria*	Antioxidant, anti-inflammatory, antihypertensive; antiviral; prevents osteoporosis	[2,60,61,62,63]
Gouda cheese	*Lactococcus cremoris* *Lactococcus lactis* *Lc. lactis subsp. lactis biovar diacetylactis* *Leuconostoc* spp.	Antioxidant, antihypertensive; beneficial effect on abdominal adipose	[64,65,66]
Mozzarella cheese	*Streptococcus thermophilus, Lactobacillus bulgaricus*, *Lactobacillus helveticus* *Lactococcus lactis* *Leuconostoc* lactic acid bacteria (LAB) *Enterococcus*	ACE-inhibitory	[67,68]
Gorgonzola cheese	*Streptococcus thermophilus, Lactobacillus delbrueckii* yeast *Penicillium roqueforti*	ACE-inhibitory, antimicrobial	[67,69]
Cheddar cheese	Mesophilic culture	Sustains blood lipid profile in individuals at risk of metabolic diseases	[70]
Kimchi	*Lactobacillus brevis*	Anti-inflammatory	[71]
Kimchi	*Lactiplantibacillus plantarum*	Antitumoral effects	[72]
Miso		Antihypertensive effects	[73]
Natto (fermented soya beans)	*Bacillus subtilis* var. *natto*	Prevention of osteoporosis; antibacterial, anticancer, antioxidant	[27]
Fermented soy product	*Lactobacillus helveticus* *Enterococcus faecium*	Improved total cholesterol	[74]
Cheonggukjang (fermented soya paste)	*Bacillus subtilis*, *Bacillus amyloliquefaciens*, *Rhizopus oligosporus*	Improves obesity-related parameters and gut microbiota dysbiosis	[75]
Water kefir	*L. mali*	Reduction of body weight and lipid accumulation	[76]
Sourdough bread	LAB culture *S. cerevisiae*	Reduction of gastric volume; higher fullness perception and hydrogen production	[17]
Fermented dairy and non-dairy food	Lactic acid bacteria; probiotic yeast species	Relieves gastrointestinal tract inflammation, IBD, IBS, and induction of cancer	[77]
Food containing prebiotic materials and dietary fibers	Probiotic species of bacteria	Minimizing risks of IBS, IBD, colorectal cancer	[78,79]
Probiotic beverages made from fermented fruits, vegetables, and cereals	Several species of probiotic bacteria	Diarrhoea control; regaining lost hydration, nutrition, and stabilizing gut microbiota	[80]
Palm wine/Toddy/Kallu (fermented sap of palm trees)	*Leuconostoc mesenteroides* *Lactobacillus plantarum * *Liquorilactobacillus nagelii * *Liquorilactobacillus sucicola * *Saccharomyces* sp. *Acetobacter* sp.	Antibacterial; improves eyesight and gastrointestinal tract	[81,82,83]
Dry-cured fermented sausages (e.g., salami, chorizo, Thai naem)	*Staphylococcus carnosus*, *Micrococcus*, lactic acid bacteria	Antioxidant, antimicrobial, antihypertensive (ACE-inhibitory)	[84]

**Table 2:** Classification of microbially derived bioactive components in fermented foods.

Category	Component or Metabolite That Provides Bioactivity	Health-Related Functions	Reference
Bacterial Components	Cell wall components (e.g., peptidoglycans, lipoteichoic acid)	Immune modulation, anti-inflammatory activity	[104,105]
	Enzymes /Surface proteins (incl. ESPs)	Enhance gut barrier, signal host receptors	[106,107]
	Exopolysaccharides (EPSs)	Immunomodulation, antioxidant activity, cholesterol reduction, prebiotic effect	[21]
	Viable probiotic cells	Gut microbiota balance, competitive exclusion of pathogens	[5,106]
Microbial Metabolites	Short-chain fatty acids (SCFAs) (e.g., acetate, propionate, butyrate)	Energy source for colon cells; anti-inflammatory; metabolic regulation	[108,109,110,111]
	Organic acids (e.g., lactic acid)	Pathogen inhibition, pH regulation, preservation	[5,96]
	Amino acids/Bioactive peptides	Antihypertensive, antioxidant; immunomodulatory effects	[106,112,113]
	Other small molecules (e.g., GABA, B vitamins, polyamines)	Neuromodulation, coenzyme function, cellular signaling	[114]

## References

[1] Terpou A., Sankaranarayanan A., Dhanasekaran N.A.D. (2020). Selected Ethnic Fermented foods of Greece. Fermented Food Products.

[2] Ganatsios V., Nigam P., Plessas S., Terpou A. (2021). Kefir as a Functional Beverage Gaining Momentum towards Its Health Promoting Attributes. Beverages.

[3] Terpou A., Rai A.K., Rai A.K., Singh S.P., Pandey A., Larroche C., Soccol C.R. (2022). 2—Microbial transformation for improving food functionality. Current Developments in Biotechnology and Bioengineering.

[4] Sahu S., Parija T., Panda S.K., Adebo O.A., Chinma C.E., Obadina A.O., Soares A.G., Panda S.K., Gan R.-Y. (2023). Chapter 25—Starter cultures: An insight into specific applications in flavoring and health promotion. Indigenous Fermented Foods for the Tropics.

[5] Terpou A., Papadaki A., Lappa I.K., Kachrimanidou V., Bosnea L.A., Kopsahelis N. (2019). Probiotics in food systems: Significance and emerging strategies towards improved viability and delivery of enhanced beneficial value. Nutrients.

[6] Dahiya D., Nigam P.S. (2023). Use of Characterized Microorganisms in Fermentation of Non-Dairy-Based Substrates to Produce Probiotic Food for Gut-Health and Nutrition. Fermentation.

[7] Kamada N., Seo S.U., Chen G.Y., Nunez G. (2013). Role of the gut microbiota in immunity and inflammatory disease. Nat. Rev. Immunol..

[8] Kovtonyuk L.V., McCoy K.D. (2023). Microbial metabolites and immunotherapy: Basic rationale and clinical indications. Semin. Immunol..

[9] Yang W., Cong Y. (2021). Gut microbiota-derived metabolites in the regulation of host immune responses and immune-related inflammatory diseases. Cell. Mol. Immunol..

[10] Garavaglia B., Vallino L., Amoruso A., Pane M., Ferraresi A., Isidoro C. (2024). The role of gut microbiota, immune system, and autophagy in the pathogenesis of inflammatory bowel disease: Molecular mechanisms and therapeutic approaches. Asp. Mol. Med..

[11] Dimidi E., Cox S.R., Rossi M., Whelan K. (2019). Fermented Foods: Definitions and Characteristics, Impact on the Gut Microbiota and Effects on Gastrointestinal Health and Disease. Nutrients.

[12] Balasubramanian R., Schneider E., Gunnigle E., Cotter P.D., Cryan J.F. (2024). Fermented foods: Harnessing their potential to modulate the microbiota-gut-brain axis for mental health. Neurosci. Biobehav. Rev..

[13] Hernández-Velázquez R., Flörl L., Lavrinienko A., Sebechlebská Z., Merk L., Greppi A., Bokulich N.A. (2024). The future is fermented: Microbial biodiversity of fermented foods is a critical resource for food innovation and human health. Trends Food Sci. Technol..

[14] Marco M.L., Heeney D., Binda S., Cifelli C.J., Cotter P.D., Foligné B., Gänzle M., Kort R., Pasin G., Pihlanto A. (2017). Health benefits of fermented foods: Microbiota and beyond. Curr. Opin. Biotechnol..

[15] O’Connor P.M., Kuniyoshi T.M., Oliveira R.P.S., Hill C., Ross R.P., Cotter P.D. (2020). Antimicrobials for food and feed; a bacteriocin perspective. Curr. Opin. Biotechnol..

[16] Plessas S., Mantzourani I., Alexopoulos A., Alexandri M., Kopsahelis N., Adamopoulou V., Bekatorou A. (2023). Nutritional Improvements of Sourdough Breads Made with Freeze-Dried Functional Adjuncts Based on Probiotic Lactiplantibacillus plantarum subsp. plantarum and Pomegranate Juice. Antioxidants.

[17] Rizzello C.G., Portincasa P., Montemurro M., Di Palo D.M., Lorusso M.P., De Angelis M., Bonfrate L., Genot B., Gobbetti M. (2019). Sourdough Fermented Breads are More Digestible than Those Started with Baker’s Yeast Alone: An In Vivo Challenge Dissecting Distinct Gastrointestinal Responses. Nutrients.

[18] Plessas S. (2021). Innovations in Sourdough Bread Making. Fermentation.

[19] Yilmaz B., Sharma H., Melekoglu E., Ozogul F. (2022). Recent developments in dairy kefir-derived lactic acid bacteria and their health benefits. Food Biosci..

[20] Rafique N., Jan S.Y., Dar A.H., Dash K.K., Sarkar A., Shams R., Pandey V.K., Khan S.A., Amin Q.A., Hussain S.Z. (2023). Promising bioactivities of postbiotics: A comprehensive review. J. Agric. Food Res..

[21] Zhang J., Xiao Y., Wang H., Zhang H., Chen W., Lu W. (2023). Lactic acid bacteria-derived exopolysaccharide: Formation, immunomodulatory ability, health effects, and structure-function relationship. Microbiol. Res..

[22] Kim M.J., Jeong J.Y., Hwang I.M., Lee J.-H. (2025). Modulation of fermentation dynamics in kimchi using Leuconostoc mesenteroides starter. Food Biosci..

[23] Chang H.C. (2018). Healthy and safe Korean traditional fermented foods: Kimchi and chongkukjang. J. Ethn. Foods.

[24] Park K.-Y., Jeong J.-K., Lee Y.-E., Daily J.W. (2014). Health Benefits of Kimchi (Korean Fermented Vegetables) as a Probiotic Food. J. Med. Food.

[25] Farooqui A.A., Farooqui T., Farooqui A.A. (2021). Chapter 4—Importance of fermented foods on human health. Gut Microbiota in Neurologic and Visceral Diseases.

[26] Harahap I.A., Suliburska J., Karaca A.C., Capanoglu E., Esatbeyoglu T. (2025). Fermented soy products: A review of bioactives for health from fermentation to functionality. Compr. Rev. Food Sci. Food Saf..

[27] Wang C., Chen J., Tian W., Han Y., Xu X., Ren T., Tian C., Chen C. (2023). Natto: A medicinal and edible food with health function. Chin. Herb. Med..

[28] Łepecka A., Szymański P., Okoń A., Zielińska D. (2023). Antioxidant activity of environmental lactic acid bacteria strains isolated from organic raw fermented meat products. LWT.

[29] Phong H.X., Viet L.Q., Chau L.M., Long B.H.D., Thanh N.N., Phat D.T., Truong L.D. (2023). Isolation and Selection of Lactic Acid Bacteria with the Capacity of Producing γ-aminobutyric Acid (GABA) and Antimicrobial Activity: Its Application in Fermented Meat Product. Curr. Nutr. Food Sci..

[30] Guo Q., Chen P., Chen X. (2023). Bioactive peptides derived from fermented foods: Preparation and biological activities. J. Funct. Foods.

[31] Hill C., Tancredi D.J., Cifelli C.J., Slavin J.L., Gahche J., Marco M.L., Hutkins R., Fulgoni V.L., Merenstein D., Sanders M.E. (2023). Positive Health Outcomes Associated with Live Microbe Intake from Foods, Including Fermented Foods, Assessed using the NHANES Database. J. Nutr..

[32] Dahiya D., Nigam P.S. (2022). Nutrition and Health through the Use of Probiotic Strains in Fermentation to Produce Non-Dairy Functional Beverage Products Supporting Gut Microbiota. Foods.

[33] Sharma R. (2021). Bioactive food components for managing cellular senescence in aging and disease: A critical appraisal and perspectives. PharmaNutrition.

[34] Naseem Z., Mir S.A., Wani S.M., Rouf M.A., Bashir I., Zehra A. (2023). Probiotic-fortified fruit juices: Health benefits, challenges, and future perspective. Nutrition.

[35] Kim D.H., Jeong D., Kim H., Seo K.H. (2019). Modern perspectives on the health benefits of kefir in next generation sequencing era: Improvement of the host gut microbiota. Crit. Rev. Food Sci. Nutr..

[36] Silva-Cutini M.A., Almeida S.A., Nascimento A.M., Abreu G.R., Bissoli N.S., Lenz D., Endringer D.C., Brasil G.A., Lima E.M., Biancardi V.C. (2019). Long-term treatment with kefir probiotics ameliorates cardiac function in spontaneously hypertensive rats. J. Nutr. Biochem..

[37] Iwatani S., Yamamoto N. (2019). Functional food products in Japan: A review. Food Sci. Hum. Wellness.

[38] Nascimento da Silva K., Favero A.G., Ribeiro W., Ferreira C.M., Sartorelli P., Cardili L., Bogsan C.S., Bertaglia Pereira J.N., de Cassia Sinigaglia R., Cristina de Moraes Malinverni A. (2023). Effects of kefir fermented milk beverage on sodium dextran sulfate (DSS)-induced colitis in rats. Heliyon.

[39] Santos A., San Mauro M., Sanchez A., Torres J.M., Marquina D. (2003). The antimicrobial properties of different strains of Lactobacillus spp. isolated from kefir. Syst. Appl. Microbiol..

[40] Shao Y., Wang Z., Tian X., Guo Y., Zhang H. (2016). Yeast β-d-glucans induced antimicrobial peptide expressions against Salmonella infection in broiler chickens. Int. J. Biol. Macromol..

[41] Hossain M.I., Sadekuzzaman M., Ha S.-D. (2017). Probiotics as potential alternative biocontrol agents in the agriculture and food industries: A review. Food Res. Int..

[42] Chifiriuc M.C., Cioaca A.B., Lazar V. (2011). In vitro assay of the antimicrobial activity of kephir against bacterial and fungal strains. Anaerobe.

[43] Ewaschuk J.B., Walker J.W., Diaz H., Madsen K.L. (2006). Bioproduction of conjugated linoleic acid by probiotic bacteria occurs in vitro and in vivo in mice. J. Nutr..

[44] Mobasherpour P., Yavarmanesh M., Edalatian Dovom M.R. (2024). Antitumor properties of traditional lactic acid bacteria: Short-chain fatty acid production and interleukin 12 induction. Heliyon.

[45] Zhang Q., Ding M., Huang Z., Jiang S., Zhao J., Stanton C., Ross R.P., Chen W., Yang B. (2025). *Bifidobacterium longum* subsp. Infantis modulates intestinal immunity in growing mice in a strain-specific manner. Food Biosci..

[46] Veiga P., Pons N., Agrawal A., Oozeer R., Guyonnet D., Brazeilles R., Faurie J.M., van Hylckama Vlieg J.E., Houghton L.A., Whorwell P.J. (2014). Changes of the human gut microbiome induced by a fermented milk product. Sci. Rep..

[47] Han K., Park J.S., Kim Y.-W., Lee W., Park K., Kim S.-K. (2025). Efficient surface display of single-chain variable fragments against tumor necrosis factor α on engineered probiotic Saccharomyces boulardii and its application in alleviating intestinal inflammation in vivo. New Biotechnol..

[48] Dahiya D., Terpou A., Dasenaki M., Nigam P.S. (2023). Current status and future prospects of bioactive molecules delivered through sustainable encapsulation techniques for food fortification. Sustain. Food Technol..

[49] Chen K.Y., Luo H.L., Li Y.Q., Han X.M., Gao C.C., Wang N.Y., Lu F.P., Wang H.K. (2023). TK1501 fermented soybeans alleviate dextran sulfate sodium-induced colitis by regulating intestinal cell function. J. Sci. Food Agric..

[50] Shakerian M., Razavi S.H., Ziai S.A., Khodaiyan F., Yarmand M.S., Moayedi A. (2015). Proteolytic and ACE-inhibitory activities of probiotic yogurt containing non-viable bacteria as affected by different levels of fat, inulin and starter culture. J. Food Sci. Technol..

[51] Sharma M., Chandel D., Shukla G. (2020). Antigenotoxicity and Cytotoxic Potentials of Metabiotics Extracted from Isolated Probiotic, Lactobacillus rhamnosus MD 14 on Caco-2 and HT-29 Human Colon Cancer Cells. Nutr. Cancer.

[52] Chen Y., Liu W., Xue J., Yang J., Chen X., Shao Y., Kwok L.Y., Bilige M., Mang L., Zhang H. (2014). Angiotensin-converting enzyme inhibitory activity of Lactobacillus helveticus strains from traditional fermented dairy foods and antihypertensive effect of fermented milk of strain H9. J. Dairy Sci..

[53] Guo W., Song Y., Sun Y., Li C., Du H., You Q., Cai Y., Lang Y., Shao L. (2025). Association Between Probiotic, Prebiotic, Synbiotics, and Yogurt Supplements and Diabetic Kidney Disease: The National Health and Nutrition Examination Survey 2007–2016. J. Ren. Nutr..

[54] Lim S.-M., Lee N.-K., Kim K.-T., Paik H.-D. (2020). Probiotic Lactobacillus fermentum KU200060 isolated from watery kimchi and its application in probiotic yogurt for oral health. Microb. Pathog..

[55] Uttarwar R.G., Mekonnen S.A., Van Beeck W., Wang A., Finnegan P., Roberts R.F., Merenstein D., Slupsky C.M., Marco M.L. (2024). Effects of Bifidobacterium animalis subsp. lactis BB-12 and yogurt on mice during oral antibiotic administration. Microbiol. Res..

[56] Hata Y., Yamamoto M., Ohni M., Nakajima K., Nakamura Y., Takano T. (1996). A placebo-controlled study of the effect of sour milk on blood pressure in hypertensive subjects. Am. J. Clin. Nutr..

[57] Mizushima S., Ohshige K., Watanabe J., Kimura M., Kadowaki T., Nakamura Y., Tochikubo O., Ueshima H. (2004). Randomized controlled trial of sour milk on blood pressure in borderline hypertensive men. Am. J. Hypertens..

[58] Yu L., Han X., Cen S., Duan H., Feng S., Xue Y., Tian F., Zhao J., Zhang H., Zhai Q. (2020). Beneficial effect of GABA-rich fermented milk on insomnia involving regulation of gut microbiota. Microbiol. Res..

[59] Tillisch K., Labus J., Kilpatrick L., Jiang Z., Stains J., Ebrat B., Guyonnet D., Legrain–Raspaud S., Trotin B., Naliboff B. (2013). Consumption of Fermented Milk Product With Probiotic Modulates Brain Activity. Gastroenterology.

[60] Prado M.R., Blandon L.M., Vandenberghe L.P., Rodrigues C., Castro G.R., Thomaz-Soccol V., Soccol C.R. (2015). Milk kefir: Composition, microbial cultures, biological activities, and related products. Front. Microbiol..

[61] Hamida R.S., Shami A., Ali M.A., Almohawes Z.N., Mohammed A.E., Bin-Meferij M.M. (2021). Kefir: A protective dietary supplementation against viral infection. Biomed. Pharmacother..

[62] Tu M.-Y., Han K.-Y., Chang G.R.-L., Lai G.-D., Chang K.-Y., Chen C.-F., Lai J.-C., Lai C.-Y., Chen H.-L., Chen C.-M. (2020). Kefir Peptides Prevent Estrogen Deficiency-Induced Bone Loss and Modulate the Structure of the Gut Microbiota in Ovariectomized Mice. Nutrients.

[63] Tiss M., Souiy Z., Abdeljelil N.b., Njima M., Achour L., Hamden K. (2020). Fermented soy milk prepared using kefir grains prevents and ameliorates obesity, type 2 diabetes, hyperlipidemia and Liver-Kidney toxicities in HFFD-rats. J. Funct. Foods.

[64] Higurashi S., Kunieda Y., Matsuyama H., Kawakami H. (2007). Effect of cheese consumption on the accumulation of abdominal adipose and decrease in serum adiponectin levels in rats fed a calorie dense diet. Int. Dairy J..

[65] Decadt H., De Vuyst L. (2023). Insights into the microbiota and defects of present-day Gouda cheese productions. Curr. Opin. Food Sci..

[66] Álvarez Ramos L., Arrieta Baez D., Dávila Ortiz G., Carlos Ruiz Ruiz J., Manuel Toledo López V. (2022). Antioxidant and antihypertensive activity of Gouda cheese at different stages of ripening. Food Chem. X.

[67] Smacchi E., Gobbetti M. (1998). Peptides from several italian cheeses inhibitory to proteolytic enzymes of lactic acid bacteria, pseudomonas fluorescens ATCC 948 and to the angiotensin I-converting enzyme. Enzym. Microb. Technol..

[68] Guidone A., Zotta T., Matera A., Ricciardi A., De Filippis F., Ercolini D., Parente E. (2016). The microbiota of high-moisture mozzarella cheese produced with different acidification methods. Int. J. Food Microbiol..

[69] Morandi S., Silvetti T., Battelli G., Brasca M. (2019). Can lactic acid bacteria be an efficient tool for controlling Listeria monocytogenes contamination on cheese surface? The case of Gorgonzola cheese. Food Control.

[70] Feeney E.L., Barron R., Dible V., Hamilton Z., Power Y., Tanner L., Flynn C., Bouchier P., Beresford T., Noronha N. (2018). Dairy matrix effects: Response to consumption of dairy fat differs when eaten within the cheese matrix—A randomized controlled trial. Am. J. Clin. Nutr..

[71] Choi C.Y., Kim Y.H., Oh S., Lee H.J., Kim J.H., Park S.H., Kim H.J., Lee S.J., Chun T. (2017). Anti-inflammatory potential of a heat-killed Lactobacillus strain isolated from Kimchi on house dust mite-induced atopic dermatitis in NC/Nga mice. J. Appl. Microbiol..

[72] Lee H.A., Kim H., Lee K.W., Park K.Y. (2016). Dietary Nanosized Lactobacillus plantarum Enhances the Anticancer Effect of Kimchi on Azoxymethane and Dextran Sulfate Sodium-Induced Colon Cancer in C57BL/6J Mice. J. Environ. Pathol. Toxicol. Oncol..

[73] Yoshinaga M., Toda N., Tamura Y., Terakado S., Ueno M., Otsuka K., Numabe A., Kawabata Y., Uehara Y. (2012). Japanese traditional miso soup attenuates salt-induced hypertension and its organ damage in Dahl salt-sensitive rats. Nutrition.

[74] Cardoso Umbelino Cavallini D., Jovenasso Manzoni M.S., Bedani R., Roselino M.N., Celiberto L.S., Vendramini R.C., De Valdez G.F., Saes Parra Abdalla D., Aparecida Pinto R., Rosetto D. (2016). Probiotic Soy Product Supplemented with Isoflavones Improves the Lipid Profile of Moderately Hypercholesterolemic Men: A Randomized Controlled Trial. Nutrients.

[75] Cha Y.-S., Edward O., Mun E.-G. (2023). P31-011-23 Cheonggukjang Improves Obesity Related Parameters and Gut Microbiota Dysbiosis in High Fat Diet Induced Obese Mice. Curr. Dev. Nutr..

[76] Chen H., McGowan E.M., Ren N., Lal S., Nassif N., Shad-Kaneez F., Qu X., Lin Y. (2018). Nattokinase: A Promising Alternative in Prevention and Treatment of Cardiovascular Diseases. Biomark. Insights.

[77] Tubaro F., Preedy V.R. (2009). 43—Antioxidant Activity of Beer’s Maillard Reaction Products: Features and Health Aspects. Beer in Health and Disease Prevention.

[78] Renaud S., de Lorgeril M. (1992). Wine, alcohol, platelets, and the French paradox for coronary heart disease. Lancet.

[79] Castaldo L., Narváez A., Izzo L., Graziani G., Gaspari A., Di Minno G., Ritieni A. (2019). Red Wine Consumption and Cardiovascular Health. Molecules.

[80] Plessas S. (2022). Advancements in the Use of Fermented Fruit Juices by Lactic Acid Bacteria as Functional Foods: Prospects and Challenges of Lactiplantibacillus (Lpb.) plantarum subsp. plantarum Application. Fermentation.

[81] Ojo O.C., Agboola S.A. (2019). Antibacterial Effects of Palm Wine (Elaeis guineensis) on Salmonella typhi Isolated from Different Sources. Int. J. Pathog. Res..

[82] Amoa-Awua W.K., Sampson E., Tano-Debrah K. (2007). Growth of yeasts, lactic and acetic acid bacteria in palm wine during tapping and fermentation from felled oil palm (*Elaeis guineensis*) in Ghana. J. Appl. Microbiol..

[83] Prathiviraj R., Rajeev R., Jose C.M., Begum A., Selvin J., Kiran G.S. (2022). Fermentation microbiome and metabolic profiles of Indian palm wine. Gene Rep..

[84] Stadnik J., Kęska P. (2015). Meat and fermented meat products as a source of bioactive peptides. Acta Sci. Pol. Technol. Aliment..

[85] Allwood J.G., Wakeling L.T., Bean D.C. (2021). Fermentation and the microbial community of Japanese koji and miso: A review. J. Food Sci..

[86] Plessas S. (2022). The Rendering of Traditional Fermented Foods in Human Diet: Distribution of Health Benefits and Nutritional Benefits. Fermentation.

[87] Basa K., Papanikolaou S., Dimopoulou M., Terpou A., Kallithraka S., Nychas G.J.E. (2022). Trials of Commercial-and Wild-Type Saccharomyces cerevisiae Strains under Aerobic and Microaerophilic/Anaerobic Conditions: Ethanol Production and Must Fermentation from Grapes of Santorini (Greece) Native Varieties. Fermentation.

[88] Kallis M., Sideris K., Kopsahelis N., Bosnea L., Kourkoutas Y., Terpou A., Kanellaki M. (2019). Pistacia terebinthus resin as yeast immobilization support for alcoholic fermentation. Foods.

[89] Mantzourani I., Terpou A., Alexopoulos A., Bezirtzoglou E., Plessas S. (2019). Assessment of ready-to-use freeze-dried immobilized biocatalysts as innovative starter cultures in sourdough bread making. Foods.

[90] Gialleli A.I., Ganatsios V., Terpou A., Kanellaki M., Bekatorou A., Koutinas A.A., Dimitrellou D. (2017). Technological development of brewing in domestic refrigerator using freeze-dried raw materials. Food Technol. Biotechnol..

[91] Engels W., Düsterhöft E.-M., McSweeney P.L.H., McNamara J.P. (2022). Starter Cultures for Cheese Manufacture. Encyclopedia of Dairy Sciences.

[92] Laranjo M., Potes M.E., Elias M. (2019). Role of Starter Cultures on the Safety of Fermented Meat Products. Front. Microbiol..

[93] Capozzi V., Garofalo C., Chiriatti M.A., Grieco F., Spano G. (2015). Microbial terroir and food innovation: The case of yeast biodiversity in wine. Microbiol. Res..

[94] Aggarwal S., Kathuria D., Singh N. (2025). Nutritional and health promoting properties of traditional regional foods: Harnessing omics techniques for microbial and metabolite identification. J. Funct. Foods.

[95] Alongi M., Anese M. (2021). Re-thinking functional food development through a holistic approach. J. Funct. Foods.

[96] Punia Bangar S., Suri S., Trif M., Ozogul F. (2022). Organic acids production from lactic acid bacteria: A preservation approach. Food Biosci..

[97] Meisel H., Bernard H., Fairweather-Tait S., FitzGerald R., Hartmann R., Lane C., McDonagh D., Teucher B., Wal J. (2001). Nutraceutical and functional food ingredients for food and pharmaceutical applications. Br. J. Nutr..

[98] Jiang R., Zhang P., Wu X., Wang Y., Rehman T., Yao X., Luo Y., Yang Z. (2021). Expression of antimicrobial peptide Cecropin P1 in Saccharomyces cerevisiae and its antibacterial and antiviral activity in vitro. Electron. J. Biotechnol..

[99] Govindarajan D.K., Kandaswamy K. (2023). Antimicrobial peptides: A small molecule for sustainable healthcare applications. Med. Microecol..

[100] Chai K.F., Voo A.Y.H., Chen W.N. (2020). Bioactive peptides from food fermentation: A comprehensive review of their sources, bioactivities, applications, and future development. Compr. Rev. Food Sci. Food Saf..

[101] Yao L., Liu Q., Lei Z., Sun T. (2023). Development and challenges of antimicrobial peptide delivery strategies in bacterial therapy: A review. Int. J. Biol. Macromol..

[102] Terpou A., Ganatsios V., Kanellaki M., Koutinas A.A. (2020). Entrapped psychrotolerant yeast cells within pine sawdust for low temperature wine making: Impact on wine quality. Microorganisms.

[103] Ferreira I.M.P.L.V.O., Pinho O., Vieira E., Tavarela J.G. (2010). Brewer’s Saccharomyces yeast biomass: Characteristics and potential applications. Trends Food Sci. Technol..

[104] Fioriti F., Rifflet A., Gomperts Boneca I., Zugasti O., Royet J. (2024). Bacterial peptidoglycan serves as a critical modulator of the gut-immune-brain axis in Drosophila. Brain Behav. Immun..

[105] Zhang L., Liu J., Kong S., Chen N., Hung W.-L., Zhao W., Zeng Z., Zhang J., Yang Z. (2023). Lipoteichoic acid obtained from Lactobacillus paracasei via low-temperature pasteurization alleviates the macrophage inflammatory response by downregulating the NF-κB signaling pathway. J. Funct. Foods.

[106] Ağagündüz D., Karakuş G., Ozogul F., Rocha J.o.M., Bartkiene E. (2025). Chapter 12—The food-gut-health axis of dairy lactic acid bacteria. Handbook of Sourdough Microbiota and Fermentation.

[107] Hajialibabaei R., Sayeli F.G., Aghadavod E., Poudineh M., Khaledi A., Bamneshin K. (2025). The Beneficial Role of Probiotics and Gut Microbiota in Signaling Pathways, Immunity, Apoptosis, Autophagy, and intestinal barrier for Effective Wound Healing Post-Burn Injury. Microb. Pathog..

[108] Yi C., Huang S., Zhang W., Guo L., Xia T., Huang F., Yan Y., Li H., Yu B. (2025). Synergistic interactions between gut microbiota and short chain fatty acids: Pioneering therapeutic frontiers in chronic disease management. Microb. Pathog..

[109] Ma H., Yu Z., Zhao Y., Li L., Liu Y., Liu Y. (2022). Goat milk fermented with combined lactic acid bacterium alter microbial community structures and levels of the targeted short-chain fatty acids in the large intestine of mice. Food Res. Int..

[110] Sasaki H., Hayashi K., Imamura M., Hirota Y., Hosoki H., Nitta L., Furutani A., Shibata S. (2023). Combined resistant dextrin and low-dose Mg oxide administration increases short-chain fatty acid and lactic acid production by gut microbiota. J. Nutr. Biochem..

[111] Balmori V., Marnpae M., Kamonsuwan K., Chusak C., Nungarlee U., Sivapornnukul P., Chanchaem P., Payungporn S., Charoensiddhi S., Suantawee T. (2024). Comparative effects of non-fermented and Lacticaseibacillus paracasei-fermented pomelo juice on gut microbiota composition and short-chain fatty acid production: An in vitro colonic model. Food Chem. X.

[112] Ajayeoba T.A., Ijabadeniyi O.A., Montet D., Ray R.C., De Carvalho Azevedo V.A., Paramithiotis S. (2023). 9—Lactic acid bacteria for the generation of bioactive peptides. Lactic Acid Bacteria as Cell Factories.

[113] Möller N.P., Scholz-Ahrens K.E., Roos N., Schrezenmeir J. (2008). Bioactive peptides and proteins from foods: Indication for health effects. Eur. J. Nutr..

[114] Icer M.A., Sarikaya B., Kocyigit E., Atabilen B., Çelik M.N., Capasso R., Ağagündüz D., Budán F. (2024). Contributions of Gamma-Aminobutyric Acid (GABA) Produced by Lactic Acid Bacteria on Food Quality and Human Health: Current Applications and Future Prospects. Foods.

[115] Liang Q., Zhou W., Peng S., Liang Z., Liu Z., Zhu C., Mou H. (2025). Current status and potential of bacteriocin-producing lactic acid bacteria applied in the food industry. Curr. Res. Food Sci..

[116] Saud S., Xiaojuan T., Fahad S. (2024). The consequences of fermentation metabolism on the qualitative qualities and biological activity of fermented fruit and vegetable juices. Food Chem. X.

[117] Gawai K.M., Mudgal S.P., Prajapati J.B., Shah N.P. (2017). Chapter 3—Stabilizers, Colorants, and Exopolysaccharides in Yogurt. Yogurt in Health and Disease Prevention.

[118] Pimentel T.C., Cruz A.G., Pereira E., Almeida da Costa W.K., da Silva Rocha R., Targino de Souza Pedrosa G., Rocha C.d.S., Alves J.M., Alvarenga V.O., Sant’Ana A.S. (2023). Postbiotics: An overview of concepts, inactivation technologies, health effects, and driver trends. Trends Food Sci. Technol..

[119] Qayyum N., Shuxuan W., Yantin Q., Ruiling W., Wang S., Ismael M., Lü X. (2023). Characterization of Short-chain fatty acid-producing and cholesterol assimilation potential probiotic Lactic acid bacteria from Chinese fermented rice. Food Biosci..

[120] Sonnenburg E.D., Sonnenburg J.L. (2019). The ancestral and industrialized gut microbiota and implications for human health. Nat. Rev. Microbiol..

[121] Rothschild D., Weissbrod O., Barkan E., Kurilshikov A., Korem T., Zeevi D., Costea P.I., Godneva A., Kalka I.N., Bar N. (2018). Environment dominates over host genetics in shaping human gut microbiota. Nature.

[122] Jha A.R., Davenport E.R., Gautam Y., Bhandari D., Tandukar S., Ng K.M., Fragiadakis G.K., Holmes S., Gautam G.P., Leach J. (2018). Gut microbiome transition across a lifestyle gradient in Himalaya. PLoS Biol..

[123] Smits S.A., Leach J., Sonnenburg E.D., Gonzalez C.G., Lichtman J.S., Reid G., Knight R., Manjurano A., Changalucha J., Elias J.E. (2017). Seasonal cycling in the gut microbiome of the Hadza hunter-gatherers of Tanzania. Science.

[124] David L.A., Maurice C.F., Carmody R.N., Gootenberg D.B., Button J.E., Wolfe B.E., Ling A.V., Devlin A.S., Varma Y., Fischbach M.A. (2014). Diet rapidly and reproducibly alters the human gut microbiome. Nature.

[125] Wastyk H.C., Fragiadakis G.K., Perelman D., Dahan D., Merrill B.D., Yu F.B., Topf M., Gonzalez C.G., Van Treuren W., Han S. (2021). Gut-microbiota-targeted diets modulate human immune status. Cell.

[126] Gille D., Schmid A., Walther B., Vergeres G. (2018). Fermented Food and Non-Communicable Chronic Diseases: A Review. Nutrients.

[127] Villarreal-Soto S.A., Bouajila J., Pace M., Leech J., Cotter P.D., Souchard J.P., Taillandier P., Beaufort S. (2020). Metabolome-microbiome signatures in the fermented beverage, Kombucha. Int. J. Food Microbiol..

[128] Taylor B.C., Lejzerowicz F., Poirel M., Shaffer J.P., Jiang L., Aksenov A., Litwin N., Humphrey G., Martino C., Miller-Montgomery S. (2020). Consumption of Fermented Foods Is Associated with Systematic Differences in the Gut Microbiome and Metabolome. mSystems.

[129] den Hartigh L.J. (2019). Conjugated Linoleic Acid Effects on Cancer, Obesity, and Atherosclerosis: A Review of Pre-Clinical and Human Trials with Current Perspectives. Nutrients.

[130] Diaz-Lopez A., Bullo M., Martinez-Gonzalez M.A., Corella D., Estruch R., Fito M., Gomez-Gracia E., Fiol M., Garcia de la Corte F.J., Ros E. (2016). Dairy product consumption and risk of type 2 diabetes in an elderly Spanish Mediterranean population at high cardiovascular risk. Eur. J. Nutr..

[131] Chen M., Sun Q., Giovannucci E., Mozaffarian D., Manson J.E., Willett W.C., Hu F.B. (2014). Dairy consumption and risk of type 2 diabetes: 3 cohorts of US adults and an updated meta-analysis. BMC Med..

[132] Eussen S.J., van Dongen M.C., Wijckmans N., den Biggelaar L., Oude Elferink S.J., Singh-Povel C.M., Schram M.T., Sep S.J., van der Kallen C.J., Koster A. (2016). Consumption of dairy foods in relation to impaired glucose metabolism and type 2 diabetes mellitus: The Maastricht Study. Br. J. Nutr..

[133] Iwasa M., Aoi W., Mune K., Yamauchi H., Furuta K., Sasaki S., Takeda K., Harada K., Wada S., Nakamura Y. (2013). Fermented milk improves glucose metabolism in exercise-induced muscle damage in young healthy men. Nutr. J..

[134] Unno T., Choi J.H., Hur H.G., Sadowsky M.J., Ahn Y.T., Huh C.S., Kim G.B., Cha C.J. (2015). Changes in human gut microbiota influenced by probiotic fermented milk ingestion. J. Dairy Sci..

[135] Silva K.R., Rodrigues S.A., Filho L.X., Lima A.S. (2009). Antimicrobial activity of broth fermented with kefir grains. Appl. Biochem. Biotechnol..

[136] Friques A.G., Arpini C.M., Kalil I.C., Gava A.L., Leal M.A., Porto M.L., Nogueira B.V., Dias A.T., Andrade T.U., Pereira T.M. (2015). Chronic administration of the probiotic kefir improves the endothelial function in spontaneously hypertensive rats. J. Transl. Med..

[137] Klippel B.F., Duemke L.B., Leal M.A., Friques A.G., Dantas E.M., Dalvi R.F., Gava A.L., Pereira T.M., Andrade T.U., Meyrelles S.S. (2016). Effects of Kefir on the Cardiac Autonomic Tones and Baroreflex Sensitivity in Spontaneously Hypertensive Rats. Front. Physiol..

[138] Gomez-Guzman M., Toral M., Romero M., Jimenez R., Galindo P., Sanchez M., Zarzuelo M.J., Olivares M., Galvez J., Duarte J. (2015). Antihypertensive effects of probiotics Lactobacillus strains in spontaneously hypertensive rats. Mol. Nutr. Food Res..

[139] Liu C.F., Tung Y.T., Wu C.L., Lee B.H., Hsu W.H., Pan T.M. (2011). Antihypertensive effects of Lactobacillus-fermented milk orally administered to spontaneously hypertensive rats. J. Agric. Food Chem..

[140] Rodriguez-Figueroa J.C., Gonzalez-Cordova A.F., Astiazaran-Garcia H., Vallejo-Cordoba B. (2013). Hypotensive and heart rate-lowering effects in rats receiving milk fermented by specific Lactococcus lactis strains. Br. J. Nutr..

[141] An S.Y., Lee M.S., Jeon J.Y., Ha E.S., Kim T.H., Yoon J.Y., Ok C.O., Lee H.K., Hwang W.S., Choe S.J. (2013). Beneficial effects of fresh and fermented kimchi in prediabetic individuals. Ann. Nutr. Metab..

[142] Han K., Bose S., Wang J.H., Kim B.S., Kim M.J., Kim E.J., Kim H. (2015). Contrasting effects of fresh and fermented kimchi consumption on gut microbiota composition and gene expression related to metabolic syndrome in obese Korean women. Mol. Nutr. Food Res..

[143] Graham D.B., Xavier R.J. (2020). Pathway paradigms revealed from the genetics of inflammatory bowel disease. Nature.

[144] Kanai T., Mikami Y., Hayashi A. (2015). A breakthrough in probiotics: Clostridium butyricum regulates gut homeostasis and anti-inflammatory response in inflammatory bowel disease. J. Gastroenterol..

[145] Sheehan D., Moran C., Shanahan F. (2015). The microbiota in inflammatory bowel disease. J. Gastroenterol..

[146] Aden K., Rehman A., Waschina S., Pan W.H., Walker A., Lucio M., Nunez A.M., Bharti R., Zimmerman J., Bethge J. (2019). Metabolic Functions of Gut Microbes Associate With Efficacy of Tumor Necrosis Factor Antagonists in Patients With Inflammatory Bowel Diseases. Gastroenterology.

[147] Frank D.N., St Amand A.L., Feldman R.A., Boedeker E.C., Harpaz N., Pace N.R. (2007). Molecular-phylogenetic characterization of microbial community imbalances in human inflammatory bowel diseases. Proc. Natl. Acad. Sci. USA.

[148] Sugimoto M., Watanabe T., Takaoka M., Suzuki K., Murakami T., Murakami N., Sumikawa S. (2021). Anti-Inflammatory Effect on Colitis and Modulation of Microbiota by Fermented Plant Extract Supplementation. Fermentation.

[149] Martini G.R., Tikhonova E., Rosati E., DeCelie M.B., Sievers L.K., Tran F., Lessing M., Bergfeld A., Hinz S., Nikolaus S. (2023). Selection of cross-reactive T cells by commensal and food-derived yeasts drives cytotoxic T(H)1 cell responses in Crohn’s disease. Nat. Med..

[150] Sidhartha Ranjit Sinha S.U. (2024). Effects of a Fermented Food-Supplemented on Patients with Ulcerative Colitis.

[151] Lei G., Khan A., Budryn G., Grzelczyk J. (2025). Probiotic products from laboratory to commercialization. Trends Food Sci. Technol..

[152] Plessas S., Nouska C., Mantzourani I., Kourkoutas Y., Alexopoulos A., Bezirtzoglou E. (2017). Microbiological Exploration of Different Types of Kefir Grains. Fermentation.

[153] Plessas S., Alexopoulos A., Voidarou C., Stavropoulou E., Bezirtzoglou E. (2011). Microbial ecology and quality assurance in food fermentation systems. The case of kefir grains application. Anaerobe.

[154] Ribera C., Sánchez-Ortí J.V., Clarke G., Marx W., Mörkl S., Balanzá-Martínez V. (2024). Probiotic, prebiotic, synbiotic and fermented food supplementation in psychiatric disorders: A systematic review of clinical trials. Neurosci. Biobehav. Rev..

[155] SaeidiFard N., Djafarian K., Shab-Bidar S. (2020). Fermented foods and inflammation: A systematic review and meta-analysis of randomized controlled trials. Clin. Nutr. ESPEN.

[156] de Simone C. (2019). The Unregulated Probiotic Market. Clinical Gastroenterol. Hepatol..

[157] FAO/WHO (2001). Report of a Joint FAO/WHO Expert Consultation on Evaluation of Health and Nutritional Properties of Probiotics in Food including Powder Milk with Live Lactic Acid Bacteria.

[158] Bond J. (2022). Gut reactions. New Sci..

[159] Cunningham M., Vinderola G., Charalampopoulos D., Lebeer S., Sanders M.E., Grimaldi R. (2021). Applying probiotics and prebiotics in new delivery formats—Is the clinical evidence transferable?. Trends Food Sci. Technol..

[160] Zhang M., Li X., Xiao Y., Cai R., Pan X., Hu Y. (2025). Effects of a new compound probiotic on growth performance, antioxidant capacity, intestinal health, gut microbiota and metabolites of broilers. Poult. Sci..

[161] Francis D.V., Dahiya D., Gokhale T., Nigam P.S. (2024). Sustainable packaging materials for fermented probiotic dairy or non-dairy food and beverage products: Challenges and innovations. AIMS Microbiol..

